# A constitutive relationship for jointed rock mass considering the change of roughness and its application

**DOI:** 10.1038/s41598-025-31449-5

**Published:** 2025-12-23

**Authors:** Xiaolin Li, Ge Zhang, Xinyi Chen, Chao Yang

**Affiliations:** 1 Anhui and Huaihe River Institute of Hydraulic Research, Hefei, China; 2 Anhui Construction Engineering Quality Supervision and Inspection Station Co., Ltd, Hefei, China; 3 Anhui Provincial Key Laboratory of Water Science and Intelligent Water Conservancy, Water, Hefei, China; 4 School of Civil Engineering and Architecture, Three Gorges University, Yichang, 443002 Hubei China; 5 School of Civil Engineering, Anhui Vocational and Technical College, Hefei, 230011 China

**Keywords:** Rock joint, Constitutive model, Roughness, Slope stability, Deformation characteristics, Civil engineering, Natural hazards

## Abstract

The mechanical behaviors of rock joints plays a vital role in controlling the stability of layered rock slopes. Most existing constitutive models for jointed rock masses assume a constant initial roughness, ignoring its evolution during shearing and leading to inaccurate descriptions of shear deformation. To address this limitation, a new constitutive model for jointed rock masses is proposed, which explicitly considers the dynamic evolution of joint surface roughness based on the stress–displacement characteristics obtained from direct shear tests. The proposed model quantitatively links shear displacement with joint roughness degradation, providing a more accurate representation of shear stiffness evolution under loading. The proposed joint model was implemented into the numerical software and validated using laboratory shear test data under different normal stresses, showing close agreement in both peak and residual strength. Furthermore, slope stability simulations demonstrated that this model can realistically reproduce the progressive failure process and yield higher accuracy in predicting safety factors than the traditional Mohr–Coulomb joint model. The results confirm that incorporating roughness evolution enhances the reliability of numerical analysis for the deformation and failure of layered rock slopes.

## Introduction

The jointed rock mass is a complex geological material composed of numerous discontinuities that strongly influence its mechanical and hydraulic behavior^1^. The existence of these joints makes the rock mass more prone to deformation and seepage^2,3^, significantly weakening its overall strength and stiffness compared with intact rock. Consequently, jointed rock masses exhibit mechanical responses that differ markedly from those of individual rock blocks^4^. Previous studies have shown that shear deformation along joint surfaces is one of the primary mechanisms leading to deformation and failure in rock engineering, exerting a direct impact on the stability of rock slopes and underground structures^5–11^.

Extensive studies have been conducted to investigate the shear behavior of jointed rock surfaces, with direct shear testing being the most widely used experimental approach. Depending on in-situ conditions, the normal loading is typically categorized as constant normal load (CNL), constant normal stiffness (CNS), or dynamic normal load (DNL). Zandarin et al.^12^ performed shear tests on sawtooth joints with different undulation angles, complemented by numerical simulations, and confirmed the significant effect of roughness on joint strength. Similarly, Zhang et al.^13^ revealed that failure of rock masses with multiple non-persistent joints involves complex bridging modes controlled by joint configuration and stress conditions. Wang et al.^14^ further showed through triaxial single-shear tests that lateral confinement enhances joint shear strength. Jahanian et al.^15^ emphasized the crucial role of normal stress in the shear behavior of filled joints. Subsequent investigations under CNS and dynamic loading conditions have provided deeper insights into the shear behavior and strength degradation mechanisms of rough rock joints. Lee et al.^16^ investigated how normal stiffness, initial normal stress, and roughness affect frictional resistance, normal deformation, and shear response. Indraratna et al.^17,18^ analyzed the shear behavior of clay- and graphite-filled sawtooth joints, identifying pronounced low-friction effects. Mirzaghorbanali et al.¹⁹ studied cyclic shear behavior under CNS conditions and proposed a mathematical model describing strength degradation. Niktabar et al.^20^ conducted cyclic shear tests on gypsum-based joints, observing a gradual reduction in shear strength with increasing cycle count. Dang et al.^21^ examined the variation of shear force, apparent friction coefficient, and normal deformation under periodic normal loads, while Gong et al.²² characterized the macroscopic and microscopic mechanisms, energy evolution, and progressive failure behavior of discontinuous joints subjected to combined shear and sinusoidal normal loading. Yuan et al.^22^ further confirmed through dynamic direct shear tests that undulating joint interfaces display distinct shear responses under varying normal stress amplitudes.

Based on the results of indoor direct shear tests, numerous theoretical models for joint shear strength have been proposed. Seidel et al.^24^ developed a theoretical model for predicting the shear characteristics of soft rock joints based on a series of direct shear tests results under constant normal stiffness conditions. Li et al.^25^ developed an analytical model for predicting the shear stress–displacement relationship of jointed rock masses under CNS conditions, emphasizing the use of measurable physical parameters rather than empirical fitting constants. Xie et al.^26^ introduced a quantitative analysis method based on shear stress difference to determine the yield point, integrating statistical damage theory to develop a new rock joint damage constitutive model. Liu et al.^27^ proposed a new statistical damage joint constitutive model, which was capable of accurately reflecting the sharp decline in shear stress after reaching peak strength. Wang et al.^29^ conducted a series of direct shear tests on jointed rock masses with varying roughness and normal stress levels, and subsequently established a cohesive–frictional elastoplastic constitutive model for joint surfaces. Kang et al.^29^ proposed a shear strength model for jointed rock filled with clay-rich materials considering moisture content. In addition to static loading, the jointed rock masses in nature would also be subjected to dynamic loading such as explosions and earthquakes^30,31^. Bai et al.^32^ examined the influence of normal loading frequency and disturbance amplitude on the shear response of rough joints under dynamic normal load (DNL) conditions and established a new shear strength criterion for flat joint surfaces. Zhang et al.^33^ developed a joint constitutive model that accounts for the effects of cyclic normal loading and unloading. Sanei et al.^34^ compiled extensive CNL direct-shear tests on natural discontinuities and derived empirical peak-shear strength relations that explicitly couple normal stress with joint roughness, providing design-ready parameters for layered formations. Although these theoretical and empirical models have improved the prediction of joint shear behavior under various loading conditions, they primarily focus on macroscopic responses and overlook the micro-mechanical processes associated with surface morphology degradation. To better understand these microstructural changes, Jiang et al.^35^ performed direct shear experiments under both constant normal load (CNL) and constant normal stiffness (CNS) conditions, measuring the joint surfaces before and after shearing using a 3D laser scanning profilometer. Hong et al. ^36^ further explored the influence of normal stress levels and asperity scales on roughness mobilization through joint shear tests, while Park et al.^37^ and Sanei et al.^38^ introduced quantitative parameters for evaluating joint surface roughness. Although these studies have revealed the general trend of roughness variation during shear, a quantitative formulation describing the evolution of joint surface roughness has not yet been established. To date, no constitutive model for jointed rock masses has been reported that explicitly considers the evolution of roughness during the loading process.

While these theoretical models have improved the understanding of joint shear behavior at the macroscopic level, they remain limited in describing the underlying micro-mechanical mechanisms. To bridge this gap, the discrete element method (DEM) has been increasingly used to simulate the complete deformation and failure process of jointed rock masses, offering a direct means to analyze contact evolution and asperity degradation. This numerical approach allows direct consideration of microstructural features such as asperity geometry, contact evolution, and bond breakage, providing valuable insights into the mechanical behavior of jointed rock systems. Shang et al.^39^ developed a three-dimensional particle-based DEM model that incorporates the geometry of rock bridges to study the influence of bridge shape and shear velocity on the mechanical response of jointed rock masses. Saadat et al.^40^ employed a new bonded-contact model to perform a series of constant normal stiffness (CNS) direct shear simulations, analyzing the effects of surface roughness, asperity angle, and initial normal stress on shear behavior. Bahaddini et al.^41^ used PFC2D to investigate the scale effect of joint roughness on shear characteristics. Liu et al.^42^ examined pre-peak cyclic shear behavior and failure mechanisms of jointed rock masses under static CNL conditions via DEM simulations. Wang et al.^43^ applied the Voronoi algorithm to develop a grain-based model (GBM) that incorporates realistic mineral grain geometries, and Francisco et al.^44^ constructed a DEM simulation framework using digitized joint surfaces obtained through optical scanning to investigate the effects of joint roughness and asperity strength on shear performance. For multi-cavern hydropower layouts, cavern–cavern interaction constrains the allowable spacing through pillar stability considerations; using thin-pillar buckling relations and DEM, spacing criteria were derived for the Bakhtiary HPP layout^45^. These numerical studies demonstrate that DEM can effectively reproduce the complex shear deformation and failure processes of jointed rock masses, providing a powerful tool for analyzing the influence of roughness evolution on shear strength and mechanical behavior.

The above studies indicate that the roughness of joint surfaces significantly affects the mechanical properties of jointed rock mass. However, existing constitutive models for joint surfaces only consider the effect of initial roughness, neglecting the evolution of roughness during the loading process. To address this issue, this paper proposes a constitutive model for jointed rock mass that accounts for the dynamic variation of roughness during loading. The model is then embedded into the numerical software. Subsequently, a series of direct shear numerical simulations under different normal stress conditions are conducted and compared with experimental data for validation. Finally, discrete element numerical simulations of the stability of stratified rock slopes are performed, and the results are analyzed in comparison with the built-in constitutive model for jointed rock mass.

## The joint rock constitutive model considering the change of roughness during loading

**Fig. 1:**
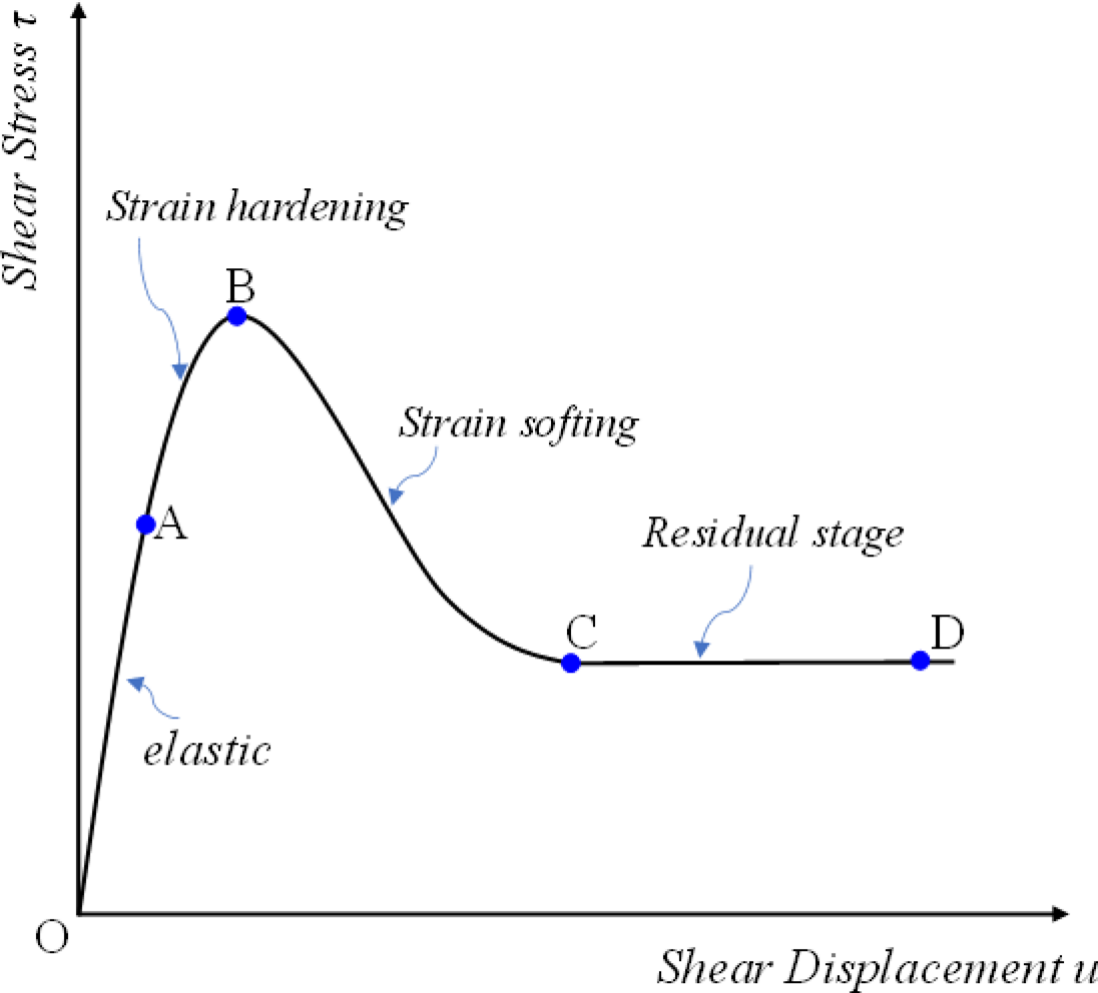
Typical shear stress-displacement curve of joint rock mass.

Figure 1 illustrates a typical direct shear force-displacement curve for jointed rock masses. The curve can be roughly divided into four stages: Elastic stage(OA), where no damage occurs at the structural plane and microasperities undergo elastic deformation; Strain hardening stage (AB), where plastic deformation of microasperities enhances load-bearing capacity, primarily due to roughness contribution; Strain softening stage(BC), where rock mass structural failure of the rock mass leads to strength degradation; Residual strength stage(CD), where steady-state frictional sliding occurs after the shear plane forms. It can be assumed that the initial roughness corresponds to the undamaged microasperities of the structural plane. The stable frictional sliding corresponds to another roughness. The roughness values at the initial and final stages must therefore differ.

This study proposed that from point A to point B, roughness increases due to the combined effect of increasing shear force and plastic deformation of microprotrusions. Subsequently, it decreases as microprotrusions undergo shear failure, eventually reaching a stable state. Therefore, the roughness of the joint surface is considered to undergo dynamic evolution during the shearing process. Such roughness variations reflect changes in microprotrusion interlocking, which in turn induce fluctuations in joint plane shear stiffness. It is therefore essential to establish equations describing roughness evolution and the governing laws of shear stiffness degradation.

### Equation for the dynamic evolution of roughness

Barton^46^ used Joint Roughness Coefficient (JRC) to characterize the geometric features of joints. The direct shear tests of jointed rock mass were conducted with different JRC values. An empirical formula for the peak shear strength of rough joint surfaces under low or moderate normal stress has been proposed:

1 $${\tau _p}={\sigma _n}\tan \left( {JRC \cdot \lg \frac{{JCS}}{{{\sigma _n}}}+{\varphi _r}} \right)$$

where JCS represents the joint wall compressive strength, and ${\varphi _r}$ is the residual friction angle. For unweathered joint plane, the JCS value equals the uniaxial compressive strength of the rock block(${\sigma _c}$). The ${\varphi _r}$ is expressed by the basic friction angle(${\varphi _b}$). The numerical value of ${\varphi _b}$ is is obtained through incline testing methods on specimens with plane joints (roughness = 0).

When there is a certain bonding strength on the joint plane, the shear strength of the joint plane is given by:

2 $${\tau _{\hbox{max} }}={\sigma _n}\tan \left( {JRC \cdot \lg \frac{{JCS}}{{{\sigma _n}}}+{\varphi _r}} \right)+{c_s}$$

where ${c_s}$ represents the cohesion of the joint surface.

Based on the typical shear force-displacement curve of joint plane, it is assumed that the roughness of joint plane follows a camel-back curve^35^, expressed as:

3 $$JRC=JR{C_0}\left( {\frac{{{S_1}u_{d}^{p}}}{{{{\left( {{S_1}+{S_2}u_{d}^{p}} \right)}^2}}}{\mathrm{+}}1} \right)$$

where $JC{S_0}$ is the initial roughness, and $u_{d}^{p}$ represents the cumulative shear displacement after the initial yielding point.

**Fig. 2:**
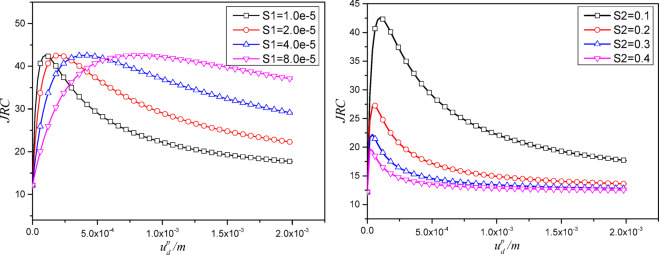
Parameter sensitivity analysis.

By conducting parameter sensitivity analysis on dimensionless parameters ${S_1}$and${S_2}$ (Fig. 2.), it can be observed that the “calculated roughness” exhibits a camel-shaped trend with cumulative shear displacement. As ${S_1}$ increases, the initial growth rate of “calculated roughness” becomes greater in the initial stage, and the residual value decreases. However, the S1 has no effect on the peak value of “calculated cohesion”. As ${S_2}$ increases, the peak value of “calculated cohesion” decreases, and the residual value also decreases. S1 controls the initial intensity of asperity crushing; increasing S1 results in a higher and delayed JRC peak with a slower approach to residual, indicating stronger interlocking and more gradual degradation. In contrast, increasing S2 lowers the peak JRC, brings it earlier, and accelerates the decay, indicating that S2 controls a smoothing length scale that governs how quickly the surface regularizes.

### Constitutive model of jointed rock

When the shear stress on the joint plane is less than the initial shear strength(${\tau _s} \leqslant {\tau _{\hbox{max} }}$), and the normal stress on the joint plane is less than the tensile strength(${\sigma _{\mathrm{n}}} \leqslant {T_n}$), the increments of shear stress and normal stress are calculated using the following formulas:

4 $$\Delta {\tau _s}={k_s}\Delta {u_s}$$

5 $$\Delta {\sigma _n}={k_n}\Delta {u_n}$$

When the shear stress on the joint plane exceeds the shear strength($\tau _{s} > \tau _{{\max }}$), the shear stress must be corrected according to the shear yield criterion. The correction formula is:

6 $${\tau _s}={\tau _{\hbox{max} }}={\sigma _n}\tan \left( {JRC \cdot \lg \frac{{JCS}}{{{\sigma _n}}}+{\varphi _r}} \right)+{c_s}$$

At the same time, the magnitude of shear plastic displacement increasement is accumulated by:

7 $$u_{d}^{p}{\mathrm{+=}}\Delta {u_s}$$

Then, the joint plane roughness is updated by Eq. (3) using the obtained shear plastic displacement. The joint plane can withstand certain tensile loads. When the normal stress on the joint plane exceeds the tensile strength$\sigma _{n} > T_{n}$, it is considered that the joint plane is completely separated, and both shear stress and normal stress are equal to zero.

From the above analysis, it can be seen that the proposed joint constitutive model includes the following parameters: normal stiffness(${k_n}$), initial shear stiffness(${k_s}$), initial roughness($JR{C_0}$), roughness evolution parameters (${S_1}$、${S_2}$), tensile strength(${T_n}$), and compressive strength of the joint wall(JCS).

## Numerical simulation of jointed rock mass direct shear

### Model development and parameter calibration

**Fig. 3:**
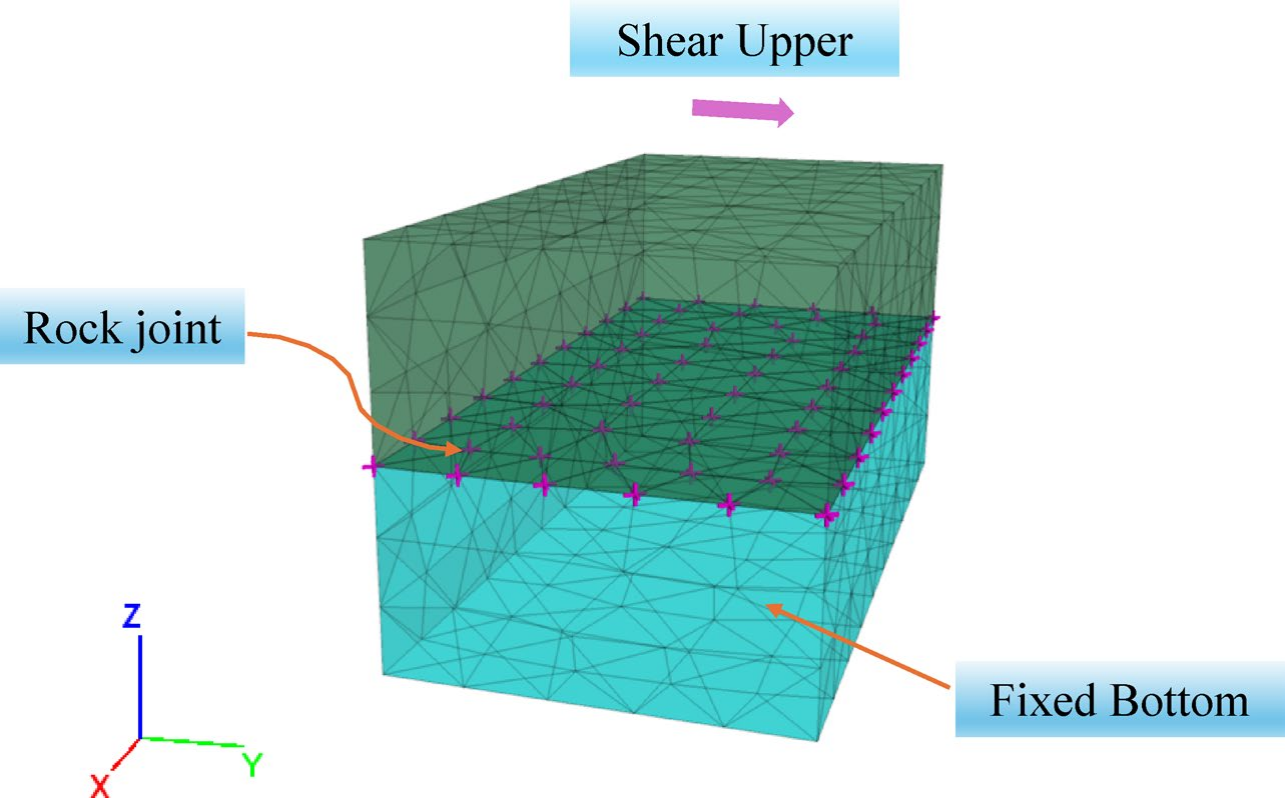
numerical simulation model.

To enable engineering applications, the proposed constitutive model of jointed rock, considering roughness variations during loading was embedded into numerical software. To verify the applicability of the proposed constitutive model for jointed rock mass, direct shear numerical simulations under different normal stresses were conducted. Referring to literature^28^, the numerical model (Fig. 3) consists of a prismatic specimen measuring 100 mm × 100 mm × 200 mm. The numerical model comprises two blocks (upper and lower). The contact interface representing the joint is assigned the proposed constitutive model. The lower part of the jointed-rock specimen is fixed. Different normal stresses (0.5 MPa, 1.0 MPa, 2.0 MPa, 3.0 MPa, 4.0 MPa) were applied to the upper surface of the specimen. Subsequently, the certain shear rate(0.2 mm/min) along the Y direction was applied to the upper part of the specimen. During the simulation, shear displacement and shear stress were recorded throughout the shearing process.

**Fig. 4:**
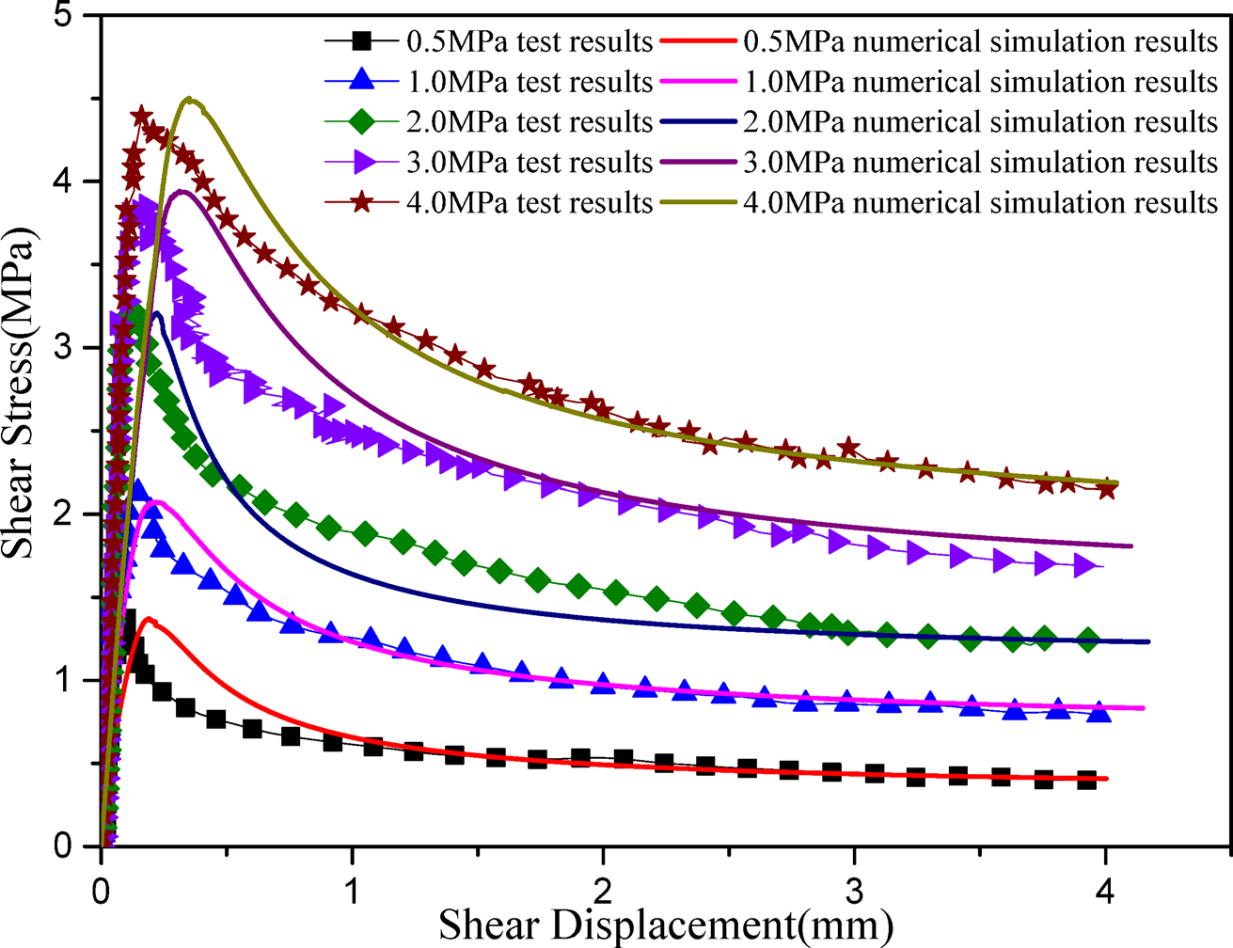
Comparison of the experimental results and the simulated results, with data from Wang et al.^28^.

Figure 4 shows a comparison between the numerical simulation results and experimental results of the shear stress-displacement curves under different normal stress conditions (JRC = 12.16). It can be seen that the proposed constitutive model accurately captures the shear mechanical properties of the joint plane, with excellent agreement between the peak and residual strengths from numerical simulations and experimental data.

**Fig. 5:**
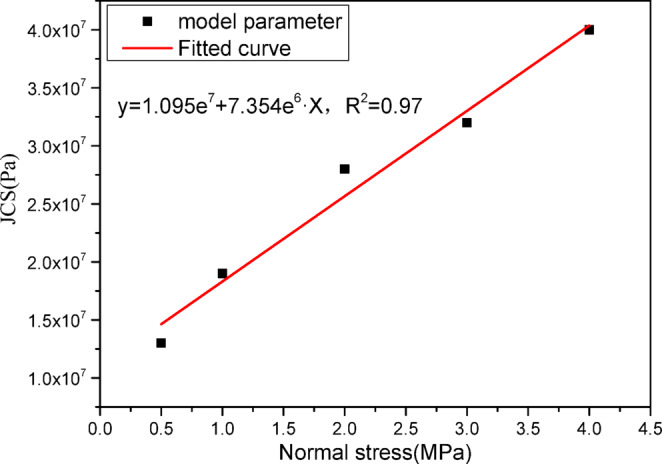
Fitting results between JCS and normal stress.

**Fig. 6:**
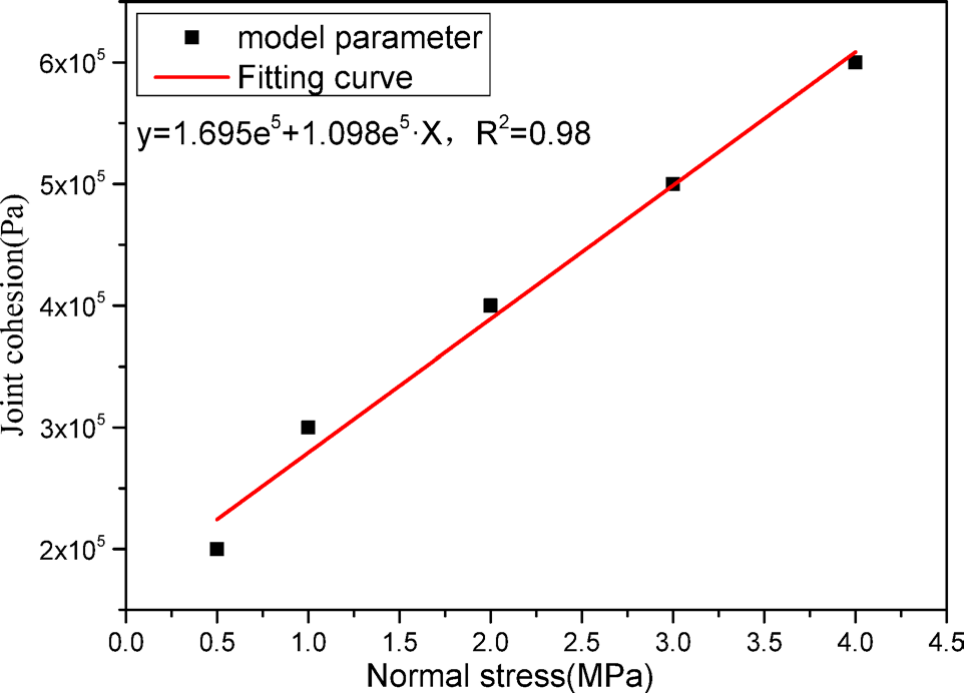
Fitting results between joint cohesion and normal stress.

The parameters of constitutive model of jointed rock are listed in Table 1. From the table, it can be seen that both the joint wall compressive strength (JCS) and joint cohesion vary with normal stress. Since only one parameter set is supplied for engineering simulations, it is necessary to fit the parameters accordingly. Figure 5 plots the joint wall compressive strength (JCS) against normal stress, and the regression indicates a linear relationship. Figure 6 shows joint cohesion versus normal stress, confirming a linear dependence. The relations fitted between joint JCS and cohesion and normal stress should be understood as effective, calibration-level parameters rather than intrinsic properties.

**Table 1:** Joint model parameters.

$\:{\sigma\:}_{n}$ (MPa)	Kn (Gpa)	Ks (Gpa)	Jrc0(/)	Jcs (MPa)	S1 (/)	S2 (/)	Cohesion (MPa)	Friction (°)	Tension(MPa)
0.5	20.0	20.0	12.6	10.0	2.0e^− 5^	0.1	0.20	10.0	2.0e^6^
1.0	20.0	20.0	12.6	19.0	2.0e^− 5^	0.1	0.30	10.0	2.0e^6^
2.0	20.0	20.0	12.6	28.0	2.0e^− 5^	0.1	0.40	10.0	2.0e^6^
3.0	20.0	20.0	12.6	32.0	2.0e^− 5^	0.1	0.50	10.0	2.0e^6^
4.0	20.0	20.0	12.6	34.0	2.0e^− 5^	0.1	0.60	10.0	2.0e^6^

**Fig. 7:**
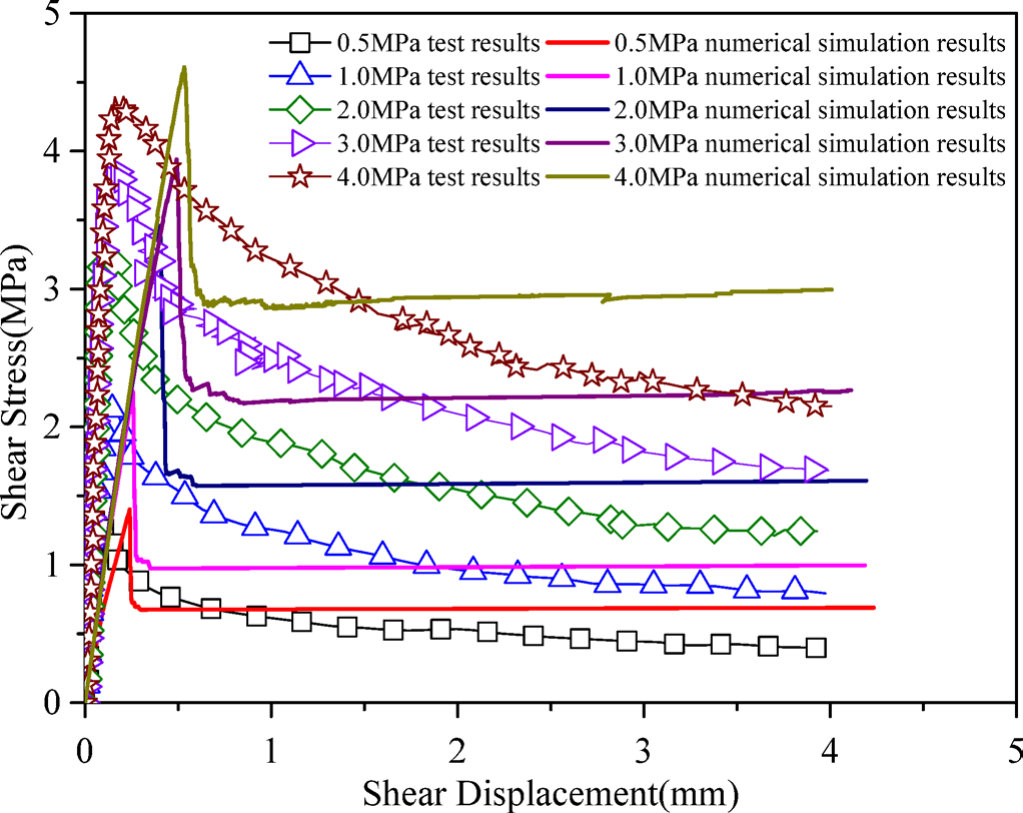
Comparison of the experimental results and the simulated results by Mohr-Coulomb joint model, with data from Wang et al.^28^.

To illustrate the value of the proposed joint model, the direct-shear simulations with a conventional Mohr–Coulomb joint model was conducted under the same geometry, boundary conditions, and loading. Figure 7 shows a comparison between the numerical and experimental shear stress-displacement curves under different normal stress conditions. It can be seen that the peak and residual strengths are in good agreement with experimental results. However, the simulations do not capture the post-peak softening of the joint shear stress–strain curves. The model parameters are shown in Table 2. ‘Cohesion-residual’ and ‘Friction-residual’ mean the residual joint cohesion, residual joint friction angle in degrees, which control the residual strength of joint.

**Table 2:** Joint model parameters of Mohr-Coulomb.

$\:{\sigma\:}_{n}$ (MPa)	Kn (Gpa)	Ks (Gpa)	Cohesion (MPa)	Friction (°)	Tension(MPa)	Cohesion-residual(MPa)	Friction-residual(°)
0.5	10.0	10.0	1.30	30.0	2.0e^6^	0.4	30
1.0	10.0	10.0	2.20	30.0	2.0e^6^	0.4	30
2.0	10.0	10.0	3.00	30.0	2.0e^6^	0.4	30
3.0	10.0	10.0	3.40	30.0	2.0e^6^	0.4	30
4.0	10.0	10.0	3.70	30.0	2.0e^6^	0.4	30

### Parameter sensitivity analysis

**Fig. 8:**
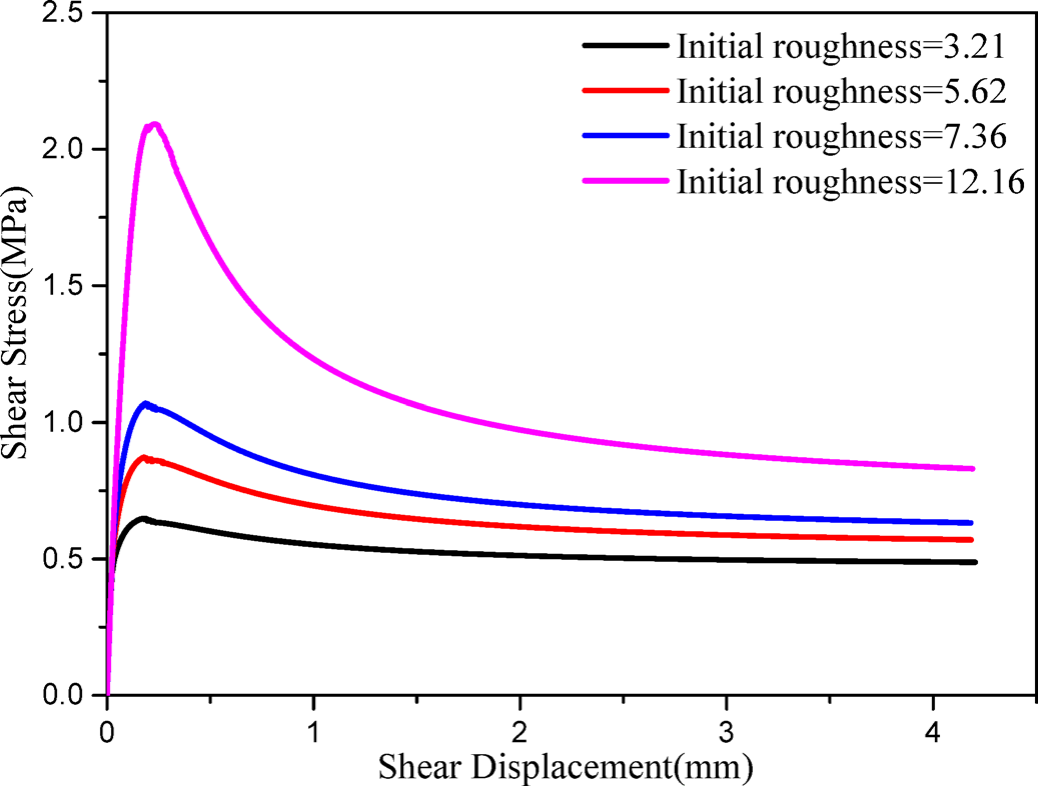
Shear stress-displacement curves for different roughness.

**Fig. 9:**
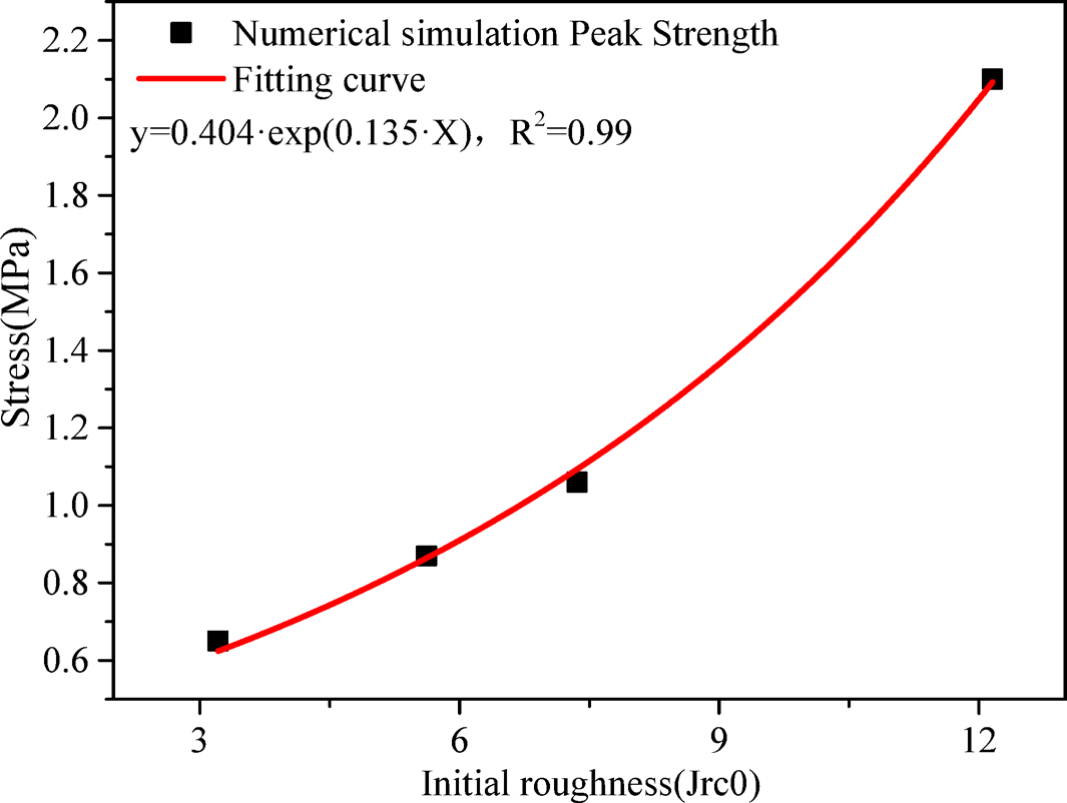
Fit curve of shear strength versus initial roughness.

As discussed in the introduction section, joint-surface roughness exerts a significant influence on the mechanical properties of jointed rock masses. Concurrently, joint roughness evolves dynamically when subjected to external loading. Accordingly, the joint constitutive model accounts for the dynamic evolution of roughness was proposed. In particular, the initial roughness of the joint surface is a critical factor governing its shear mechanical properties. It is necessary to perform a sensitivity analysis of the initial roughness. Figure 8 presents shear stress–displacement responses from direct-shear simulations at a normal stress of 1 MPa for initial roughness (Jrc0) levels 3.21, 5.62, 7.36, and 12.16. As shown, increasing the initial roughness (Jrc0) leads to higher peak and residual shear strengths, along with a more pronounced post-peak softening range. The regression in Fig. 9 indicates an exponential dependence of peak strength on roughness ($y=0.404\exp \left( {0.135 \cdot Jrc0} \right)$,${R^2}=0.99$), providing a compact mapping from the initial roughness(Jrc0) to design-level strength.

**Fig. 10:**
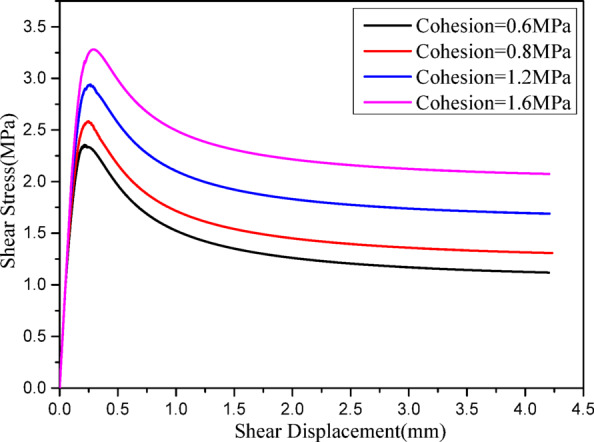
Shear stress-displacement curves for different joint cohesion.

Figure 10 presents shear stress-displacement curves under 1 MPa normal stress with different joint cohesion values (0.6 MPa, 0.8 MPa, 1.2 MPa, 1.6 MPa). It is evident that both joint shear strength and residual strength increase progressively with increasing joint cohesion.

## Stability analysis of toppling slope

**Fig. 11:**
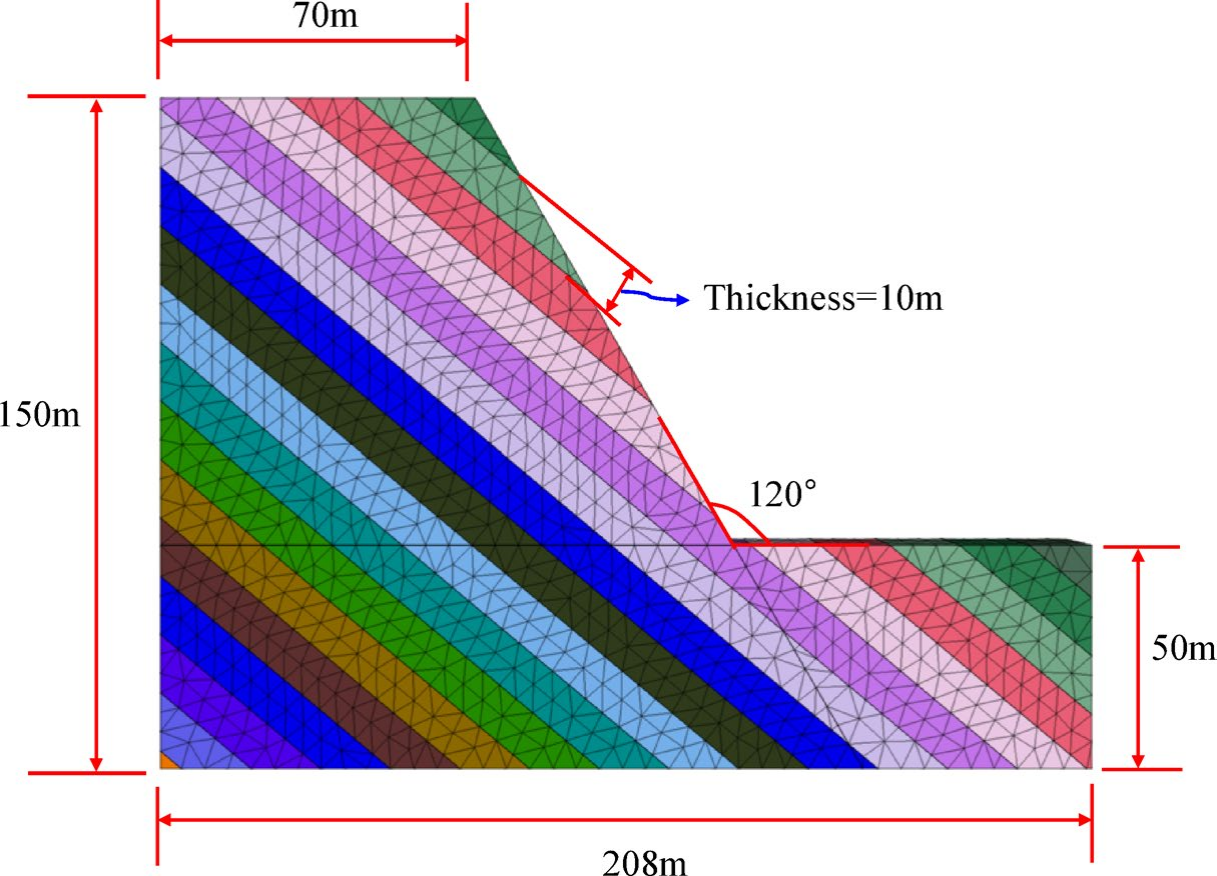
Numerical simulation calculation model.

**Fig. 12:**
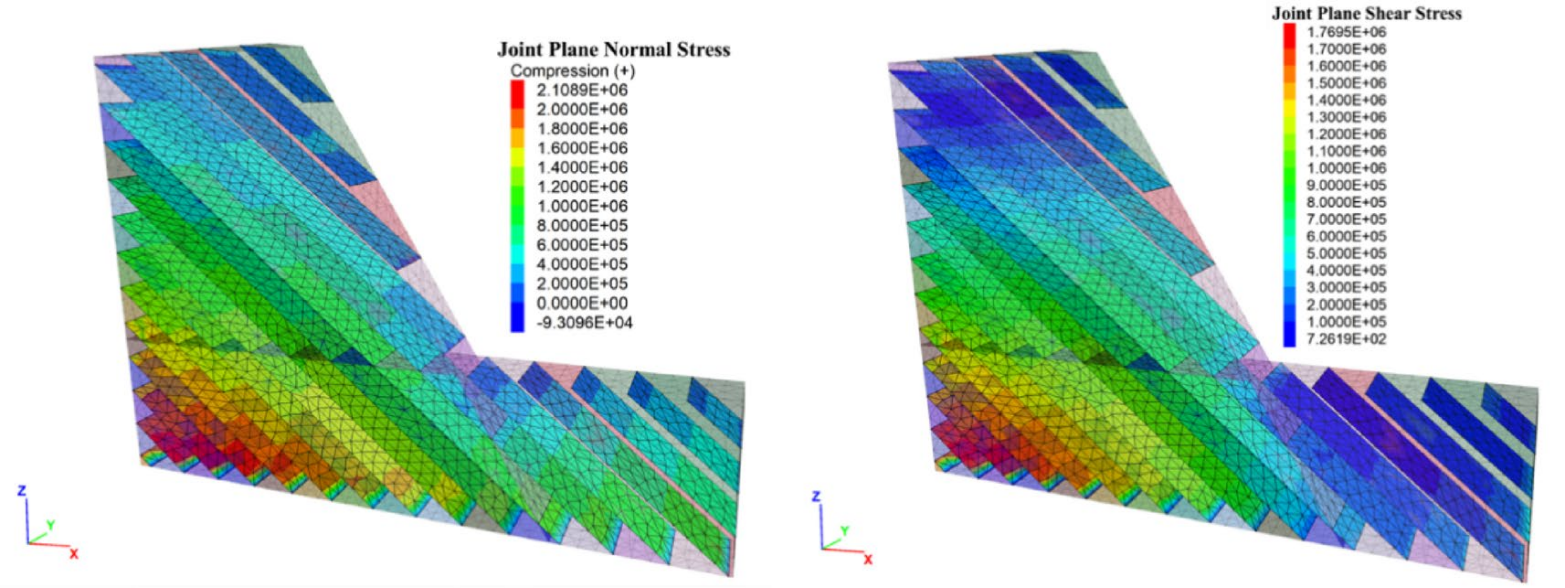
Stress state of the joint plane after initial stress equilibrium.

**Fig. 13:**
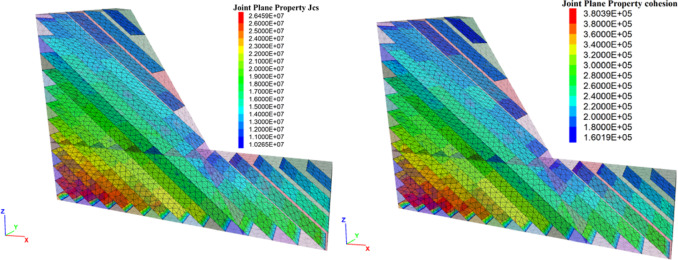
the JCS and cohesion setting.

Using the proposed joint model, a typical toppling slope stability analysis was performed. The established numerical calculation model for the inclined rock slope is shown in Fig. 11. The slope angle is selected as 60 degrees, with an interlayer thickness of 10 m. The numerical analysis of the jointed rock slope were conducted in the following steps: (1) The boundary conditions are imposed on numerical model. The boundary conditions are: bottom nodes with ${V_z}=0$, nodes on the front and back faces with ${V_y}=0$; and nodes on the left and right faces with ${V_x}=0$. (2) The proposed model was applied to the joint planes, with parameters from Table 1(JRC = 12.16). However, according to the parameter calibration in "Model development and parameter calibration", the cohesion and JCS of the joint interface vary with normal stress. First, the cohesion on the joint surface is set to an effectively infinite value so that the interface behaves elastically, after which the initial geostatic stress equilibrium is established(Fig. 12). (3) The normal stress is extracted via the FISH language, after which cohesion and JCS are assigned to the joint surfaces according to the fitted relations established in "Model development and parameter calibration" (Fig. 13). From the figure, the joint parameter JCS ranges from 1.03e^7^ to 2.64e^7^, while cohesion varies from 1.60e^5^ to 3.80e^5^. (4) Finally, the numerical slope-stability calculation is performed.

**Fig. 14:**
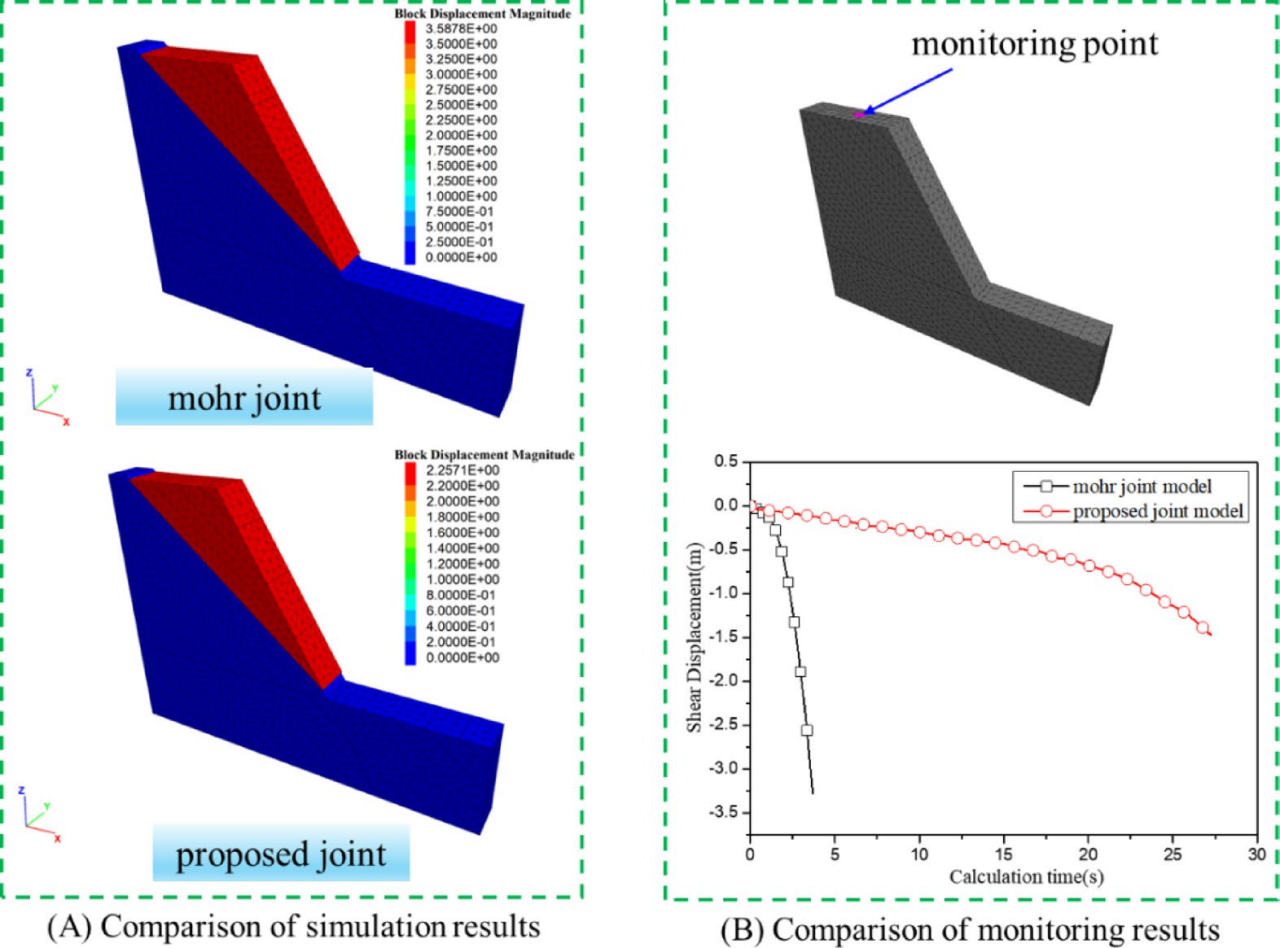
Numerical simulation results of the failure process of toppling slope.

Figure 14(A) presents the numerical simulation results of stability analysis using the proposed constitutive model and the built-in Mohr-Coulomb model. It can be seen that the sliding plane location is identical, 13 m from the left slope end. A monitoring point was placed at the top of the numerical calculation model. Figure 14(B) depicts the evolution of Z-direction displacement at the monitoring point. It is evident that when using the built-in joint model, the displacement follows a parabolic trend, whereas with the proposed model, it initially increases linearly and then accelerates.

**Fig. 15:**
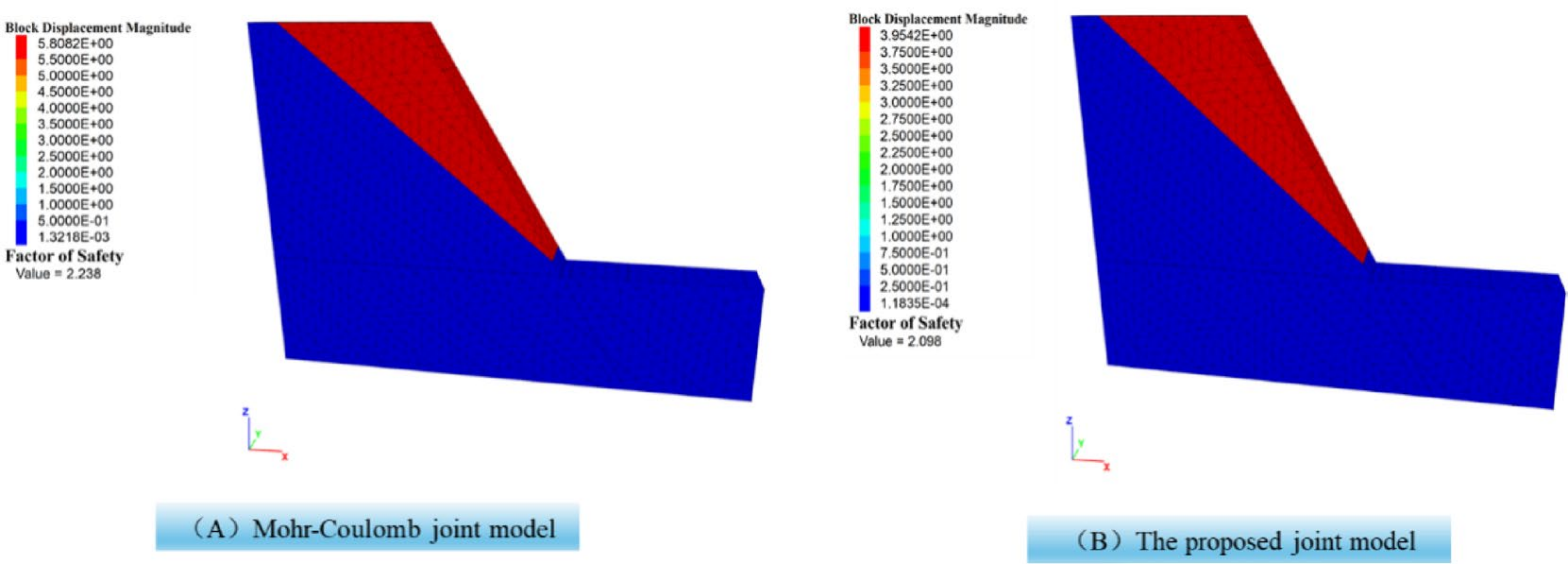
Slope stability analysis.

The slope safety factor analysis is conducted using the fitted parameters of Mohr-Coulomb. The safety factor of slope by numerical simulation is 2.098(Fig. 15(A)). The slope safety factor analysis is also conducted using the proposed joint model. The safety factor of slope by numerical simulation is 2.238(Fig. 15(B)). It can be seen that the most dangerous slip surface is the same. The safety factor obtained using the proposed joint model is higher than that of the built-in Mohr-Coulomb joint model. This is because the proposed joint model gradually softens, whereas the Mohr-Coulomb joint model undergoes brittle failure. After reaching the peak, under the same strain conditions, the proposed joint model can withstand greater stress. The difference stems from how the two joints models evolve after peak. The proposed joint model captures progressive, displacement-dependent softening of roughness. The asperity crushing and contact-area evolution reduce strength continuously rather than abruptly. At the same post-peak shear displacement, a nontrivial portion of contact patches remains partially interlocked, yielding higher mobilized shear stress. In contrast, the Mohr–Coulomb (MC) joint with fixed parameters exhibits brittle behavior. Once yielding occurs, local strength drops to a prescribed post-peaky plastic level with little displacement dependence, producing a lower average interface stress and therefore a lower computed factor of safety in this case.

**Fig. 16:**
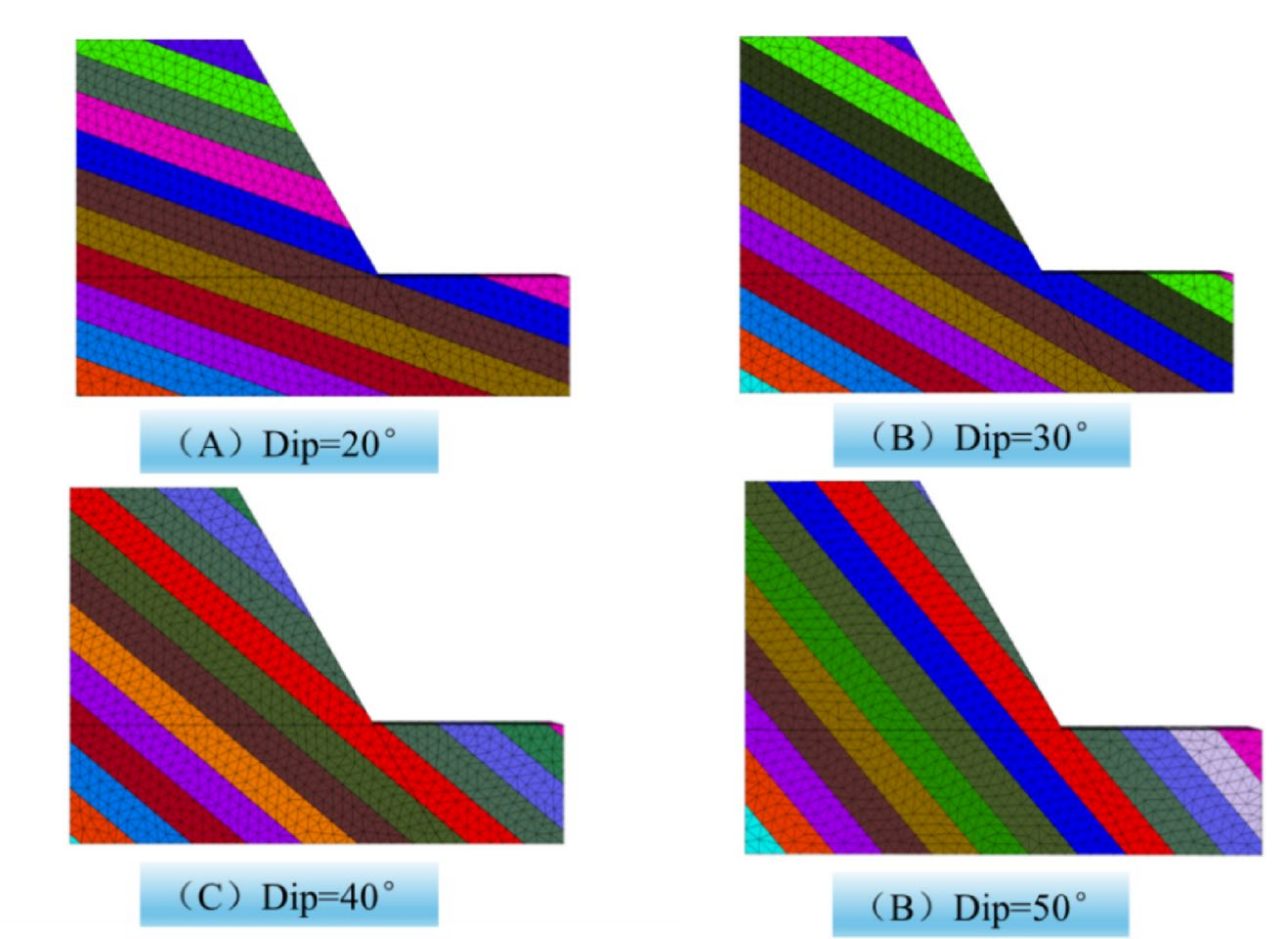
Calculation models for different joint dip.

**Fig. 17:**
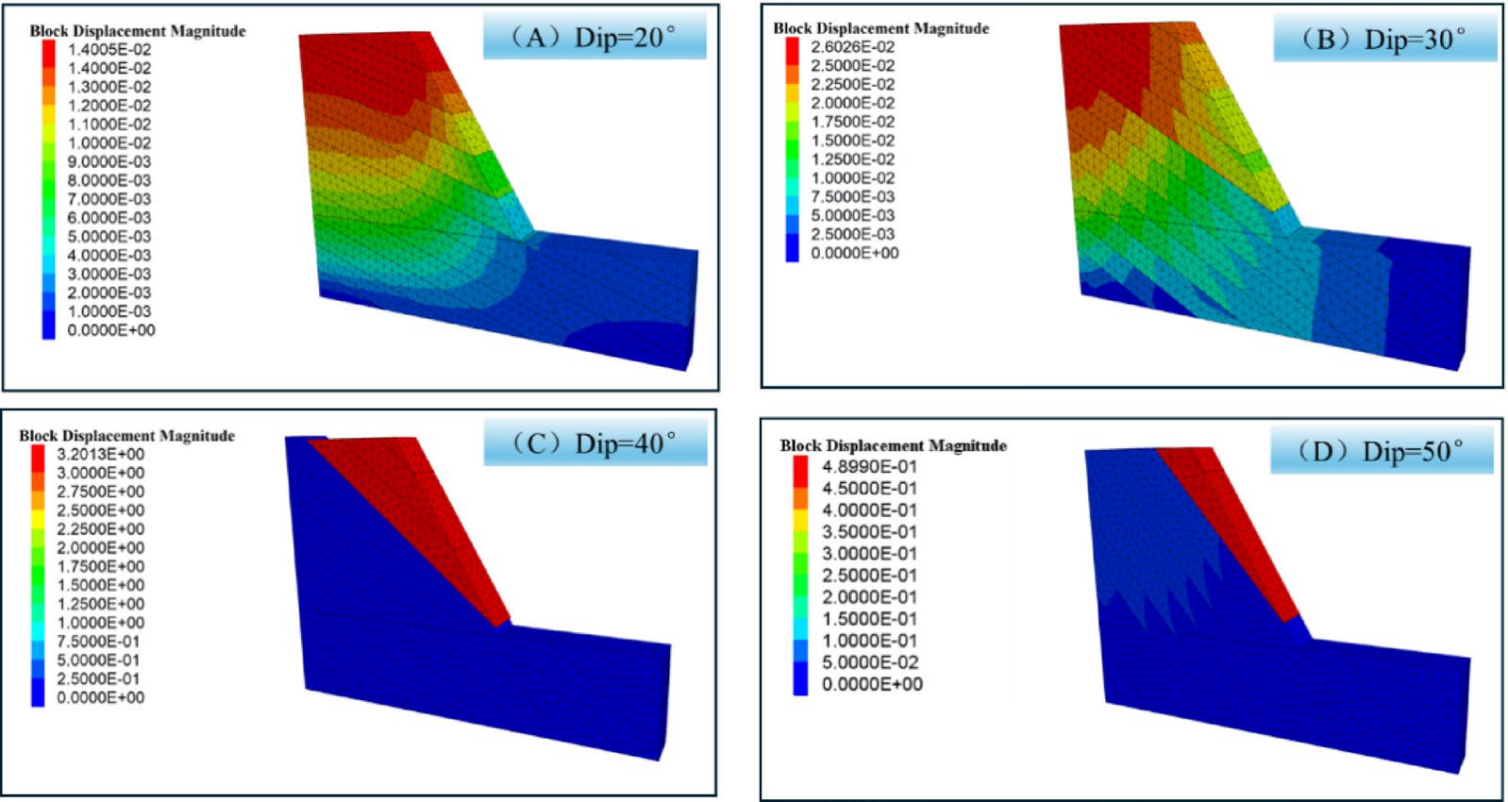
Displacement cloud map for different joint dip.

To investigate the effect of inclination on slope stability, the numerical simulations of slope stability under different joint dip conditions(20°、30°、40°、50°) were conducted. Figure 16 shows the numerical calculation models for numerical simulation. Figure 17 shows displacement cloud diagrams under different joint dip conditions. The figure shows that as the angle increases, the slope gradually becomes unstable.

## Conclusion

The constitutive model of jointed rock is proposed, which can consider the change of roughness based on the direct shear stress-displacement curve of jointed rock mass. Then the proposed model is embed into numerical software. A series of numerical simulations of direct shear tests on jointed rock masses are conducted. The results show that the proposed constitutive model accurately reflects the deformation characteristics of jointed rock masses. Subsequently, the typical rock slope stability analyses were performed. The results indicates that the proposed model can effectively simulate the failure process of rock slopes.

However, this study still has several limitations. (1) The present implementation focuses on clean and dry rock joints; the effects of infill materials, moisture or degree of saturation, gouge generation, and chemical or weathering alterations are not considered, although these factors can significantly influence joint stiffness and strength. (2) The proposed constitutive model does not account for the mechanical degradation induced by cyclic loading and unloading on the joint plane. (3) Joint roughness is simplified into a scalar index. Directional roughness, structural anisotropy, and laboratory-to-field scale effects are only indirectly represented, which may affect the accuracy of predictions in heterogeneous joint networks. (4) Parameter calibration and associated uncertainties remain important limitations. The relationships between joint wall compressive strength and cohesion versus normal stress are derived using simple linear regressions, which, although convenient, may be case-specific and not fully generalizable. (5) The pseudo-3D slope example illustrates the model’s capabilities under idealized geometric and boundary conditions. However, complex topographies and variations in joint persistence and network connectivity require further evaluation.

To address these limitations, we plan to conduct additional experiments and expand the applicability of the proposed model. Further refinements and extensions will be developed based on the present work.

## Data Availability

All data generated or analyzed during this study are included in this published article.
